# Letter to Editor: “Effect of parecoxib on postoperative delirium in patients with hyperlipidemia: a randomized, double-blind, single-center, superiority trial”

**DOI:** 10.1097/JS9.0000000000002734

**Published:** 2025-06-12

**Authors:** Yongzheng Han, Wenlong Xie, Guangzhen Wu

**Affiliations:** a Department of Urology, the First Affiliated Hospital of Dalian Medical University, Dalian, China; b Department of Ultrasound, the First Affiliated Hospital of Dalian Medical University, Dalian, China

*Dear Editor*,

We are writing to commend the recent article by Zeng Daojun *et al*, titled *“**Effect of parecoxib on postoperative delirium in patients with hyperlipidemia: a randomized, double-blind, single-center, superiority trial***”^[1]^, published in the ***International Journal of Surgery***. We hereby declare that this manuscript was translated and linguistic checked using artificial intelligence to ensure compliance with journal requirements. The TITAN Guideline checklist was submitted according to the requirements of the literature ***“Transparency In the reporting of Artificial Intelligence-The TITAN guideline”***^[2]^.

This elaborately designed and meticulously implemented study has made a significant contribution to perioperative medicine and provided new insights into the prevention of postoperative delirium (POD) in patients with hyperlipidemia. This study not only confirmed the effect of parecoxib on reducing the incidence of POD, but also clarified the potential mechanism of its protective effect, which has important clinical significance.

Through rigorous scientific research, the authors successfully demonstrated that preoperative administration of parecoxib sodium can significantly reduce the incidence of POD in patients with hyperlipidemia. Given the high prevalence of hyperlipidemia in surgical patients and the significant impact of POD on postoperative recovery and long-term cognitive function, this is a very important finding. In addition, the authors’ exploration of the mechanism of action of parecoxib is both novel and crucial. By analyzing indicators such as inflammatory cell counts and pain scores, they provided a plausible explanation for their protective effects. This effect arises from two key mechanisms: inhibition of COX-2-driven inflammation and management of postoperative pain. These insights highlight the importance of POD prevention and suggest promising treatment options. In addition, the subgroup analysis of this study identified the patient population most sensitive to parecoxib, providing a perspective for tailoring interventions to the risk profile of individual patients.

However, this excellent study also has some questions that deserve further exploration: What is the optimal timing and dose of parecoxib to prevent POD in patients with hyperlipidemia? A single preoperative administration was used in this study, but whether increasing or changing the dosing schedule can improve the efficacy is still a question worthy of further study. In addition, only patients who underwent colorectal cancer resection were included in this study. Given that POD risk varies by type of surgery, does parecoxib sodium show similar efficacy in patients with hyperlipidemia who undergo major surgery other than colorectal cancer resection? This issue seems likely to be explored further. Finally, this study assessed cognitive impairment at 3 months after surgery, but does parecoxib also protect cognition beyond 3 months after surgery in patients with hyperlipidemia. Achieving this goal may be challenging but has great clinical implications.

This study has important implications for the prevention of POD in high-risk patients. The effectiveness of parecoxib has been demonstrated, which lays a solid foundation for further research on its optimal use and mechanism of action.

## Data Availability

The data that support the findings of this study are all included in this article.

## Research registration unique identifying number (UIN)

This study did not involve human subjects and was based on retrospective data analysis/literature review. Hence, research registration was not applicable.

## References

[1] Daojun Z Yuling T Yingzhe X. Effect of parecoxib on postoperative delirium in patients with hyperlipidemia: a randomized, double-blind, single-center, superiority trial. Int J Surg Lond Engl 2025;111:2903–13. 10.1097/JS9.0000000000002286 PMC12175806 39903567

[2] Agha RA Mathew G Rashid R, Transparency in the reporting of artificial intelligence – the TITAN guideline. Prem J Sci 2025;10: 100082.

